# Implementing Chief Resident Immersion Training (CRIT) in the Care of Older Adults: Overcoming Barriers and Promoting Facilitators

**DOI:** 10.3390/geriatrics3040062

**Published:** 2018-09-26

**Authors:** Christian Davis Furman, Lori Wagner, Josephine Gomes, Rangaraj Gopalraj, B. Frank Parker, Laura Morton, Demetra Antimisiaris, Daniela Neamtu, Sadaf Masroor, Ramie Martin-Galijatovic, Samantha Cotton, M. Ann Shaw

**Affiliations:** 1 Institute for Sustainable Health & Optimal Aging, University of Louisville, 300 E. Market Street, Suite 200, Louisville, KY 40202, USA; masroorsadaf00@gmail.com (S.M.); sam.cotton@louisville.edu (S.C.); 2 Geriatrics, University of Louisville, 1941 Bishop Ln, Suite 900, Louisville, KY 40218, USA; josephine.gomes@louisville.edu (J.G.); rangaraj.gopalraj@louisville.edu (R.G.); laura.morton@louisville.edu (L.M.); demetra.antimisiaris@louisville.edu (D.A.); daniela.neamtu@louisville.edu (D.N.); 3 Division of General Internal Medicine, Palliative Medicine and Medical Education, Department of Internal Medicine, University of Louisville, 550 South Jackson St, 3rd Fl, Ste A3K00, Louisville, KY 40202, USA; lori.wagner@va.gov (L.W.); b.parker.1@louisville.edu (B.F.P.); monica.shaw@louisville.edu (M.A.S.); 4 School of Medicine, University of Louisville, Abell Administration Bldg, 323 East Chestnut St., Louisville, KY 40202, USA; 5 Robley Rex Veterans Affairs Medical Center, 800 Zorn Ave., Louisville, KY 40206, USA; 6 Kent School of Social Work, University of Louisville, Oppenheimer Hall, 2217 S. Third St., Louisville, KY 40292, USA; ramie.martingalijatovic@louisville.edu

**Keywords:** geriatrics, curriculum, older adults, interprofessional education, curricular innovations, geriatrics curriculum

## Abstract

The Chief Resident Immersion Training (CRIT) in the Care of Older Adults curriculum was developed at Boston University School of Medicine to improve the care of older adults through an educational intervention. The curriculum targeted chief residents (CRs) because their role as mediators between learners and faculty provides the greatest potential impact for transmitting knowledge. The goals of CRIT are to: (1) provide education on geriatric principles and on teaching/leadership skills, (2) foster interdisciplinary collaboration, and (3) complete an action project. This study demonstrates successful implementation of CRIT at a different academic institution in a rural state. The CRs indicated that their confidence in their ability to apply and teach geriatrics improved after CRIT. In addition, the CRs indicated that CRIT improved their confidence in their overall skills as CRs. The barriers and facilitators to implementation are addressed in order to promote successful adoption of CRIT at other institutions, including those in rural states.

## 1. Introduction

In studying and implementing educational innovations, the goal is to generalize effective programs so that they may be reproduced effortlessly in other institutions to achieve similar results and widespread adoption. After implementing the Chief Resident Immersion Training (CRIT) in the Care of Older Adults at the University of Louisville, located in a rural state, other rural states can successfully implement CRIT.

CRIT was developed by Geriatrics, General Internal Medicine, and the Department of Family Medicine at Boston University School of Medicine, after being awarded a Donald W. Reynolds Foundation grant in 2003. This funding was provided to develop the Boston University Medical Center (BMC) Comprehensive Geriatric Education project, with the intention of improving the care of older adults through education across the continuum. Chief residents were targeted because their role as mediators between medical residents/students and clinical faculty could provide the greatest potential impact for transmitting knowledge in the care of older adults [1]. To date, research has not been completed to prove the improvement of clinical outcomes after this educational intervention.

With additional funding and support,, the CRIT curriculum was implemented at 33 other institutions across the country, training over 1000 chief residents from 2007 to 2013 [2]. The goals of the CRIT conference remained the same for the additional sites: (1) to provide education on geriatric principles, (2) to provide tutelage on teaching and leadership skills, (3) to foster interdisciplinary collaboration, and (4) to create and complete an action project during the participants’ chief resident year [1]. Data from these 33 other institutions showed successful implementation nationwide.

Reporting of CRIT implementation beyond the BMC is limited in the medical literature [2,3,4,5,6,7]. There is only one published paper about CRIT and it reports on including interprofessional education into the curriculum. Only one of the presentations began to touch upon potential barriers and facilitators of successful implementation, as they presented data on their action project characteristics and completion rate [7]. The literature does not report on implementation, barriers, and facilitators in a rural state.

Implementation science is the study of translating evidence-based interventions perfected in the lab under ideal conditions into practice in real world environments, identifying explicit barriers and promoters of success [8]. Implementation science shifts the focus from collection and reporting of data to analysis and application. It can explore whether a particular intervention was successfully applied and if the barriers and the facilitators for success were addressed. Without barrier and facilitator identification, analysis, and subsequent distribution of contextual findings, similar errors in implementation will be repeated elsewhere, resulting in wasted resources. The frustration and cost, actual and opportunity, associated with suboptimal implementation may prevent incorporation of the intervention, resulting in the envisioned behavioral change never being realized. Although each context should expect to have unique barriers, a study of the barriers to and promoters of success from a different but similar institution will provide information that can be utilized in development of strategies to enhance the probability of success. This study will show that CRIT can still be implemented successfully at an institution in a rural state and will highlight the barriers and facilitators at an institution with limited resources.

This article not only presents data on CRIT implementation at the University of Louisville School of Medicine (ULSOM), but also will provide a detailed discussion of barriers to and facilitators of implementation. UL SOM differs from the BMC in many ways; therefore, other institutions seeking to implement CRIT can use this information in their implementation. The BMC is an urban medical school with 1685 medical students and 12 geriatricians. The ULSOM is located in a rural state and only has 630 medical students and five geriatricians. Even with this smaller faculty, the ULSOM was able to implement CRIT successfully. Other institutions may benefit from our experience and attempt to address barriers and implement facilitators proactively, avoiding the costs associated with suboptimal implementation.

## 2. Methods

The ULSOM was selected to receive a two-year grant to participate in CRIT. The grant was funded by the Donald W. Reynolds Foundation. As part of the grant agreement, the ULSOM agreed to use the curriculum previously developed by a multidisciplinary team of faculty from the BMC [1].

The content for the program was developed around a case presentation of an older female who presented to the emergency department with an acute abdomen and undergoes surgery. The case evolved over three 2-h interactive modules designed to foster interdisciplinary collaboration in the management of older adults.

During both 2014 and 2015, chief residents were invited from all residency programs at the ULSOM with the goal that this two-day program would enhance their knowledge about geriatric medicine, strengthen their leadership and teaching skills, and allow them opportunities to network with other chief residents. Residency program directors were also invited to attend, hoping to foster collaboration among faculty and encourage the program director to serve as a mentor to the chief resident on his/her action project. As part of the cost-sharing agreement of the grant, each program was charged $500 (USA) for the program director, two chief residents, and their families to be able to attend.

## 3. Participants

A total of 18 chief residents participated in 2014 and 10 chief residents in 2015. In 2014, most of the chief residents were Caucasian (94%), with the remainder being Asian Americans. In 2015, 60% of the participants were Caucasian, 10% were Asian American, 10% were African American, and 20% classified themselves as “Other” (Table 1). In 2014, the majority of attendees were family medicine (FM) residents. In 2015, the majority of attendees were FM and internal medicine residents. Emergency medicine, general surgery, podiatry, medicine/pediatrics, radiation oncology, and other residents attended in both years (Table 2). In 2014, 11% of chief residents indicated that they had attended a medical school outside of the United States. In 2015, none of the chief residents attended a medical school outside of the United States.

## 4. Procedures

During both years, CRIT occurred at a hotel sixty miles from Louisville during early June. An off-site location was selected to minimize resident distractions. The program began with a live patient/family interview regarding navigating the healthcare system when the patient became ill. This interview was followed by two modules on Saturday and one module on Sunday. Each module consisted of the case presentation, small group discussion, and two-three mini-lectures. Small groups consisted of chief residents and program directors, and were facilitated by the faculty. Mini-lectures included decision-making capacity, care of the hospitalized older adult, opioid use in older adults, delirium, functional assessment, polypharmacy, and discharge planning. Details of the case presentation are located in the Appendix A.

Between the main modules, mini-lectures were given on facilitation skills, techniques for giving feedback, working with the reluctant learner, and conflict resolution to enhance chief residents’ leadership and teaching skills. 

During both years, participants had time in the afternoon to spend with each other, their families and guests, and participate in a variety of recreational activities. Participants and their guests attended a reception, followed by dinner, on Saturday. 

Each chief resident was expected to develop an action project that focused on management of older patients and aligned with the chief resident’s interests and his/her residency program’s needs. During the CRIT program, two working meetings were held where each chief resident met one-on-one with faculty to develop their action project. Near the end of the conference, chief residents shared their action project with a larger group of faculty for feedback. At the end of CRIT, chief residents turned in a copy of their action project.

Chief residents completed a comprehensive survey that was sent out a month before CRIT, completed a post-CRIT survey, and completed a survey six months after the completion of CRIT. This survey was developed by the interdisciplinary team at BMC with expert consultation from a research consulting group. BMC strictly enforced that the same surveys should be used at all participating institutions. The survey asked for demographic information, about background and interest in geriatrics, about clinical practice and teaching related to the care of older patients, about skills of the chief resident, about skills in geriatrics, about the action project, and about feedback on the CRIT program. Each chief resident signed a consent form allowing the use of their data. The University of Louisville Institutional Review Board determined that this study did not meet criteria for human subjects research. 

## 5. Results

The total number of residents employed in the participating departments (programs) varied, with a mean of 21 residents/department (program). The mean number of medical students rotating through the departments (programs) per year was 73 students (with a range of 0 to 200 students). These numbers represent the impact the chief residents will have on learners in their departments (programs) after attending CRIT. Only chief residents were invited to participate in CRIT. 

Participants were asked how many hours over the last two years they participated in a variety of venues where geriatrics was the focus or topic. In 2014, the highest concentration of mean hours were attending rounds (M = Mean hours, M = 18.00; SD = 36.48), while in 2015, geriatric electives constituted the highest mean concentration (M = 30.40; SD = 67.31). The chief residents stated they had approximately 80–102 h of geriatrics exposure during their residency. 

In 2014, only about one-third of chief residents had a rotation in geriatrics during their medical school (28%) and over half during their residencies (56%). In 2015, 80% had a rotation in geriatrics during medical school, but only 50% had a rotation during their residency.

On a scale of 1–7, with 1 being “not at all” and 7 being “very much”, the mean rating was 3.89 for the degree to which training about geriatrics is addressed in their program in 2014. In 2015, the mean rating was 2.8. 

The chief residents were slightly more interested in the geriatric age groups relative to other age groups—average of 3.22 at baseline (scale 1 (a lot less)—3 (is about the same)—5 (a lot more)) compared to 3.42 at 6-month follow-up, in the 2014 cohort. In 2015, the baseline data showed an average of 2.8—leaning towards less interest in geriatric issues. However, in the 6-month follow-up of the 2015 cohort, the data showed an increase to an average of 3.44—slightly more interest in the geriatric age group.

Data analysis was conducted using non-parametric statistics due to the fact that sample size for both years of the program was small (2014 *N* = 18; 2015 = 10), and preliminary analysis showed that the data collected was not normally distributed. Table 3 shows the reported confidence of clinical practice and teaching as it relates to the care of older patients. Participants were asked about their confidence in applying clinical problem solving, ability to teach others clinical problem-solving skills related to the care of older adults, and the ability to incorporate geriatric issues into formal and informal teaching. They were also asked to rate the degree to which CRIT contributed to this improvement. For the 2014 cohort, ability to apply clinical problem-solving skills to the care of older patients increased over time. A Wilcoxon signed ranks test indicated that the median post-test (post-CRIT) ranks were statistically significantly higher than the median pre-test (pre-CRIT) ranks (z = −2.60; *p* = 0.014). The 2014 cohort also showed change from pre-CRIT to six months after CRIT in their perceived confidence to teach others clinical problem-solving skills related to the care of older patients. A Wilcoxon signed ranks test indicated that the median post-test (post-CRIT) ranks were statistically significantly higher than the median pre-test (pre-CRIT) ranks (z = −2.89; *p* = 0.004). Furthermore, the 2014 cohort showed change in terms of confidence to incorporate geriatric issues into teaching. A Wilcoxon signed ranks test indicated that the median post-test (post-CRIT) ranks were statistically significantly higher than the median pre-test (pre-CRIT) ranks (z = −2.71; *p* = 0.005). For the 2015 cohort, perceived confidence in ability to apply clinical problem-solving skills to the care of older patients showed a significant change from pre-CRIT to six months after CRIT (z = −2.67; *p* = 0.007) and confidence in ability to incorporate geriatrics issues into formal and informal teaching (z = −2.46; *p* = 0.14).

After attending CRIT, the 2015 chief resident cohort taught more geriatric medicine topics than prior to attending CRIT. Six months after CRIT, 88% of the chief residents taught about caring for hospitalized older adults (compared to 30% prior to CRIT). In addition, 88% of the chief residents taught about recognizing delirium after CRIT (compared to only 20%prior to CRIT).

The 2014 chief resident cohort also improved their geriatric medicine teaching after attending CRIT. They taught topics related to the care of older adults in bedside teaching, other small group teaching, and at other conferences/lectures after the CRIT. For the 2014 cohort, analysis using a Wilcoxon signed ranks test indicated that the median post-test ranks at six months were statistically significantly higher than the median pre-test ranks in all these categories (for bed-side teaching (z = −2.13; *p* = 0.03); for other small group teaching opportunities (z = −2.04; *p* = 0.04); and for other conferences and lectures (z = −2.81; *p* = 0.005). For the 2015 cohort, for each of these variables, the difference between pre and at the six-month time interval was not statistically significant. 

For the 2014 and 2015 cohorts, analysis using a Wilcoxon signed ranks test indicated that the median post-test ranks at six months were statistically significantly higher than the median pre-test ranks regarding the chief residents’ perception about the extent to which medical students, house staff and/or faculty come to them as a resource on geriatrics (for 2014: z = −3.23; *p* = 0.05; for 2015: z = −2.77; *p* = 0.03).

The chief residents’ surveys indicate that the instruction about giving feedback, dealing with the reluctant learner, small group facilitation skills, and conflict resolution was effective in increasing their leadership and teaching abilities (Table 4). The chief residents indicated that CRIT played a role in improving these skills (seen in “Contributed by CRIT data” in Table 4). It appears that the most effective components of the CRIT curriculum were those that provided practical skills about teaching and leadership.

The least successful part of the CRIT curriculum involved the action projects (Table 5). No residents completed their action project in 2015 and only one completed their action project in 2014. During both years, the majority of the chief resident action projects addressed resident education. 

## 6. Analyses

The ULSOM successfully implemented CRIT through a two-year grant. The surveys demonstrate an improvement in chief residents’ confidence in clinically managing older adults. In addition, teaching related to the care of older adults improved after CRIT. After CRIT, chief residents were considered a resource for medical students, house staff, and/or faculty regarding geriatrics issues. CRIT also improved the teaching and leadership skills of chief residents. During both years, chief residents appreciated the opportunity to meet and interact with chiefs from other disciplines. They stated that this was a highlight of the conference. They never had this collaborative opportunity before. Older adults receive better care when their providers know each other and communicate with each other.

The chief residents had prior exposure to geriatrics during their residency programs, but it was not consistent. If the chief residents had not attended CRIT, they would have graduated without being taught geriatrics. Geriatrics was also not consistently taught during medical school. Only 27.8% of the 2014 chief residents had a rotation in geriatrics during their medical school training, however 80% of the 2015 chief residents had a rotation in geriatrics. The increase in 2015 to 80% of chief residents being taught geriatrics in medical school is impressive and encouraging. Medical schools are starting to realize the importance of teaching geriatrics in the context of an aging population. This increase in geriatric medical education in medical school in 2015 might explain why Table 3 and 4 shows a larger improvement in 2015 data compared to 2014 data for clinical practice and teaching related to the care of older patients.

The exposure to geriatric training for medical students and residents at the ULSOM is limited, as it is at most U.S. medical school and training programs. A survey of medical schools in the United States shows that less than half of the responding schools have a structured geriatrics curriculum and that a quarter require a geriatrics clerkship [9]. Barriers commonly cited to preventing full integration of geriatrics into health science programs include: time, scarcity of educators (and lack of advocates), geriatric stereotyping (including lack of exposure to healthy aging). Some learners state that it is difficult to find locations where there is sufficient exposure to older adult patients [10].

In regards to the chief resident action projects, only one of the chief residents over the two-year period was able to complete the project. The participants met with faculty mentors during CRIT to develop initial plans for implementation of the action project. However, even with the assignment of mentors, residents and mentors did not move their projects forward to completion. Some of the chief residents cited barriers including lack of time to complete their planned project, and lack of expert geriatric mentor availability (Table 6). One faculty mentor reported that her chief resident was located on a physically different training site (the VA (Veterans Affairs) Medical Center vs. the ULSOM) and was not responsive to email updates, which implies that having an onsite geriatric faculty mentor, who can be present face-to-face for the mentee might have made a difference in persistence with the CRIT intervention. One method of overcoming this barrier would be to ensure that there is a geriatrics mentor point-person at each training site to both assist with CRIT dissemination and check-in on the progress of action projects throughout the year (Table 6).

We also seemed to have less than robust outcomes with other measures, such as the ability of chief residents to teach others clinical problem-solving skills related to the care of older adults, the amount of responsibility chief residents feel for teaching geriatric issues, and their enjoyment of teaching geriatrics. Having geriatrics faculty experts as advocates and periodically progress checking each chief resident throughout the year might create an improved outcome, especially if time is the most significant barrier. The time for the chief resident themselves is limited and their expertise in geriatrics is nascent, making their work of leading in an area where they are not expert more time consuming and difficult. More frequent check-ins with the chief residents and their residency unit, and monitoring of desired outcome implementation can help create a culture that empowers the chief residents (Table 6).

## 7. Discussion

CRIT was successfully implemented over a two-year period at the University of Louisville, located in a rural state. The chief residents who attended CRIT had a positive impact on their departments (programs) by teaching geriatrics to the residents and students. If they did not attend CRIT, they would not have had enough exposure to geriatrics in their residency. After attending CRIT, the chief residents were more interested in the geriatric age group. The chief residents had increased confidence in their clinical practice and teaching related to the care of older adults after attending CRIT. They also gave more presentations on geriatric medicine topics after attending CRIT. The chief residents also had a perception that they were a resource on geriatrics for medical students, other residents and faculty. They also had increased confidence in their overall leadership skills after CRIT. CRIT implementation in a rural state was not reported previously in the literature.

One limitation of this research is the small number of participants. We plan to offer this program to nursing graduate students, social work graduate students, and community workers in the field of geriatrics in the future. In this way, the program will be more interdisciplinary and have more participants. In the future, it was discussed that CRIT could be open to all residents. This might help with recruiting residents to the geriatric medicine fellowship and increase CRIT attendance.

Chief residents’ confidence in their leadership and teaching skills went down at six months. However, immediately after CRIT, the chief residents stated they were confident in their leadership and teaching skills. Therefore, at six months, a reunion/refresher course is needed. It would be beneficial if the chief residents bring difficult geriatric medicine cases they have experienced in the past six months in order to learn from their recent experiences.

One barrier for some departments to attend was cost. They could not afford the $500 (USA) fee. In the future, scholarships will be offered to ensure everyone who would like to attend will be able to attend (Table 6).

A positive unintended consequence of CRIT was that the Geriatric Medicine was consulted with in the hospital more frequently after CRIT. The chief residents met with the Geriatric Medicine Faculty at CRIT; therefore, they felt more comfortable in asking for assistance in caring for older adults. 

The findings in this study are applicable to other institutions who might want to implement the curriculum. Geriatric medicine is usually a small specialty at academic medical centers. If the ULSOM, located in a rural state, could implement this curriculum with limited faculty and resources, other institutions can also do so. In addition, the barriers of time and cost are universal. Awareness that these barriers need to be addressed proactively will ensure the success of CRIT. In addition, other institutions can implement the items that facilitate a successful CRIT. Hosting CRIT at a resort, allowing families to attend, protecting free time, face-to-face meetings with mentors, frequent progress checks, refresher courses at six months, and scholarships to attend CRIT are all possible with pre-planning.

In order to improve the completion rate of the action projects, another suggestion is to have a six-month follow-up meeting with all CRIT participants to report on the progress of the action projects. The faculty mentor and CRIT learner are more likely to complete the action project if they have a deadline to present the action project in front of their peers.

CRIT did accomplish some of the goals that the planners intended, but was not successful in all areas. However, the overall impact of CRIT was positive. There is a clear indication that CRIT training should continue. The chief residents have a positive impact on the faculty, residents, and students with whom they interact (some chief residents interact with up to 200 medical students per year). Geriatric medicine will now be taught to all these faculty members, residents, and students. CRIT will continue at the ULSOM and it will expand to include more of the interdisciplinary team. The hope is that older adults will receive better care because their providers are trained in geriatric medicine through CRIT. This clinical outcome will need to be studied after future CRIT conferences. Academic medical centers in rural states will now have the tools to successfully implement CRIT because they will be aware of the barriers and facilitators to implementation.

## Figures and Tables

**Table 1:** Basic demographics of learners.

Variable	2014	2015
% Female	44%	60%
% Male	56%	40%
% Caucasian	94%	60%
% Asian American	6%	10%
% African American	-	10%
% Other race/ethnicity	-	10%
Total chief residents	18	10

**Table 2:** Medical specialties of learners.

Specialty	2014	2015
Family medicine	3	3
Internal medicine	4	-
Emergency medicine	1	1
Surgery	-	1
Anesthesiology	-	1
Psychiatry	1	-
Ophthalmology	-	1
Urology	1	
Otolaryngology	2	
Radiation oncology	-	1
Other	6	2

**Table 3:** Clinical Practice and Teaching (CRIT) related to the care of older patients: Overall confidence—reported as averages; 1 = low; 5 = high; pre-CRIT and 6 months after CRIT.

Skill Set	Pre-Training 2014	6-Month Follow-Up 2014	* Denotes Significant at 0.05	Pre-Training 2015	6-Month Follow-Up 2015	* Denotes Significant at 0.05
(1) Your ability to apply clinical problem-solving skills to the care of older patients	3.78	4.0	*	3.2	4.22	*
** Contribution by CRIT		2.25			2.78	
(2) Your ability to teach others clinical problem-solving skills related to the care of older patients	3.67	3.92	*	3.1	3.67	
** Contribution by CRIT		2.17			2.56	
(3) Your ability to incorporate geriatrics issues into your formal and informal teaching	3.61	3.92	*	2.8	4.22	*
** Contribution by CRIT		2.17			2.67	

* = significant at 0.05; ** Degree to which CRIT contributed to this improvement: 1 = not at all; 2 = somewhat; 3 = a lot.

**Table 4:** Chief resident (CR) leadership skills—overall confidence—reported as averages; 1 = not at all; 5 = very much; pre-CRIT, immediately after the CRIT, and 6 months after the CRIT.

Leadership Skills	Pre-Training 2014	6-Month Follow-Up 2014	Pre-Training 2015	6 Month Follow-Up 2015
(1) Small group facilitation skills	3.67	3.58	2.7	4.11
** Contributed by CRIT		2.17		#
(2) Ability to give feedback	3.72	4.0	3.0	3.89
** Contributed by CRIT		2.17		#
(3) Ability to connect with a reluctant learner	3.39	3.5	2.8	4.33
** Contributed by CRIT		2.25		2.11
(4) Ability to teach case-based interactive approach	3.78	Not asked	2.9	Not asked
(5) Ability to resolve conflicts	3.61	3.83	3.3	#
** Contributed by CRIT		2.17		2.22
(6) Overall ability to carry out work as CR	4.06	4.08	3.7	#
** Contributed by CRIT		2.17		2.33
(7) Ability to apply clinical problem-solving skills to older patients	3.78	4.0	3.2	4.22
** Contributed by CRIT		2.25		2.78
(8) Ability to teach others clinical problems solving skills re older pts	3.67	3.91	3.1	4.22
** Contributed by CRIT		2.17		2.56
(9) Ability to incorporate geriatric issues into teaching	3.61	3.92	2.8	4.22
** Contributed by CRIT		2.17		2.67

** Degree to which CRIT contributed to this improvement: 1 = not at all; 2 = somewhat; 3 = a lot. # Incomplete data.

**Table 5:** Action projects; immediately after CRIT and 6 months after CRIT.

Action Project	Post-Training 2014 *	Post-Training 2015 *	6 Months 2014	6 Months 2015
Likelihood of carrying out action project in the next 6 months	4.0 *	3.25 *	N/A	N/A
Feeling excited about developing/carrying out my action project	4.08 *	4.0 *	N/A	N/A
Usefulness of meeting with the geriatric faculty about the action project	4.42 *	4.0 *		
Did you feel you had adequate time to develop your idea for the action project?	85% said yes	67% said yes		
Percentage of the action project complete (# of CR with that percentage)			1 = 0%, 4 = 10%, 3 = 20% 1 = 30% 1 = 50% 1 = 70% 1 = 80%	4 = 0%, 1 = 10%, 2 = 20%, 1 = 30%, 1 = 50%
Action Project Addressed:				
a. Medical student education			25%	11%
b. Resident education			67%	67%
c. Other education at my institution			33%	33%
d. Other education outside my institution			8%	11%
e. Patient safety			50%	11%
f. Patient outcomes			17%	11%
g. Quality improvement			50%	56%
h. Other				11%
Format of Action Project:				
a. Single teaching event			33%	22%
b. Multiple session teaching event			42%	11%
c. Tool/instrument or related			8%	33%
d. Information card or related			25%	33%
e. Research or analysis of existing data			16%	0%

N/A = not-applicable; * Post-training scale (2014–2015): 1 = not at all; 2 = a little; 3 = somewhat; 4 = very much; 5 = completely.

**Table 6:** Barriers and facilitators to implementation of CRIT at the University of Louisville School of Medicine (ULSOM).

Barriers		Facilitators	
1.	Lack of time in various areas: in CRIT, to complete the action project, and for the chief resident (CR) and mentor to meet and monitor the CR’s desired outcomes	1.	Assigning mentors for action projects
2.	Lack of time to attend CRIT	2.	Make CRIT attractive by hosting it at a resort hotel, allowing family members to attend, and protecting free time to relax at the hotel
3.	Scarcity of geriatricians to serve as educators	3.	All geriatricians at the institution attend CRIT; face to face meeting with mentor at each training site
4.	Lack of geriatrics mentor available on-site to meet about action project	4.	Frequent contact about action projects and geriatrics teaching and progress-checking
5.	Baseline geriatric knowledge limited in the learners	5.	Reunion/refresher course at 6 months
6.	Cost of $500 (USA) is prohibitive for some departments	6.	Provide scholarships to attend CRIT

## Appendix A. CRIT Case

**Module I:**

Mrs. HH is an 84-year-old African-American woman with the following:

BPPV—Benign paroxysmal positional vertigo
Type 2 Diabetes
HTN—Hypertension
Osteoporosis
Hypercholesterolemia
Glaucoma
Hearing Loss: Wears hearing aids bilaterally
Arthritis
Status post (S/P) total abdominal hysterectomy for fibroids

CC (Chief Complaint): Abdominal pain

HPI (History of Present Illness): Patient’s daughter visited the patient at home today and found that the patient was “not herself”. Patient complains of dizziness. She has had abdominal pain for a couple of days but thought that it was caused by something she ate. Pain comes and goes. She has decreased appetite. Eating food worsens her abdominal pain. She tried taking Mylanta to ease the pain, but this did not help. Patient does not have hematochezia, melena, fevers, chills, dysuria or back pain.

MEDS: No known drug allergies (NKDA)

- glipizide XL 10 mg daily (*type 2 diabetes*)
- metformin 500 mg twice a day (*type 2 diabetes*)
- lisinopril 10 mg daily (*hypertension*)
- hydrochlorothiazide 25 mg daily (*hypertension*)
- atorvastatin 20 mg daily (*hypercholesterolemia*)
- alendronate 70 mg weekly (*osteoporosis*)
- calcium w/vitamin D daily (*osteoporosis*)
- multivitamin daily
- latanoprost one gtt (drop) OU (both eyes) at bedtime (*glaucoma*)
- timolol one gtt OU twice a day (*glaucoma*)
- acetaminophen as needed for pain

Social History: Born in Virginia. Finished eighth grade. Married for 65 years. Husband is still alive but had a stroke 5 years ago and is very ill. Wife is primary caregiver since the stroke. He has right arm hemiparesis. Three adult children: one in California, 2 are local and are involved in the patient’s care. She worked as a home health aide all of her adult life until she retired at age 68. Attends the AME (African Methodist Episcopal) church where she used to sing in the choir. Lives in a small single-family house in a residential area of Louisville, KY. Bedroom and bathroom are both on the second floor.

The patient receives social security payments. She has Medicare Parts A, B and D. Patient’s husband used to work for University of Louisville Hospital (ULH). He receives a pension in addition to social security payments. He has Medicare Parts A and B with supplemental private health insurance benefits. They do not qualify for Medicaid benefits based on their income.

ADLs (Activities of Daily Living): Completely independent.

IADLS (Instrumental Activities of Daily Living): A homemaker comes once a week from an Area Agency on Aging (AAA) provider to do light cleaning. The AAA provides Case Management Services. Her daughter helps with shopping but the patient can still go out shopping on her own. The patient gives herself and her husband medications. Sometimes she takes her medications, sometimes not, depending on how much money she has. She cooks. She hasn’t been to see an MD in 8 months because she has been caring for her husband and doesn’t have time.

Pertinent PE (Physical exam): Physical exam in the nurse practitioners (NP) office

On arrival: BP 90/60 (normal 120/80), HR 120 (normal 60–80), T 100.7 (normal 98.6)

Moaning in pain; appears uncomfortable. Calling for daughter.

HEENT (Head, eyes, ears, nose, throat): Dry mucous membranes, flat neck veins. Hearing aids are absent.

Lungs: Clear

Abd: Absent bowel sounds (BS), +guarding, rebound left lower quadrant (LLQ)

Neuro: Moving all four extremities. Mini mental status exam (MMSE): 22/30

NP sends the patient to the Emergency Room (ER). In the ER,

Labs: Hct 34% with MCV 99, WBC 17.5 K with left shift, plts 280K

Na 145 mmol/L, K 3.2 mmol/L, Cl 105 mmol/L, CO2 20 mmol/L, AG 20 (metabolic acidosis), Glu 300 mg/dL

BUN 45 mg/dL, Creat 1.3 mg/dL; LFTs WNL, Ca/Mg, sent and pending

EKG: sinus tachycardia

Abdominal X-ray: ??thickened bowel wall LLQ, ??free air

CT abdomen → free air, stranding around L descending colon

Normal blood pressure after 1 L normal saline given

Diagnosis: Physical exam, laboratory data, and imaging data are concerning for diverticulitis and perforation.

Plan: Surgery was consulted. They recommend that she go to the operating room. When the surgeons inform the patient about their assessment and recommendations, she repeatedly states, “I can’t understand you”. The surgeons then try to get daughter to sign consent. Daughter says, “My mother makes her own decisions”. Surgeons admit patient to their service.

**Module II:**

Mrs. HH is an 84-year-old African-American woman who presented to the emergency room with dizziness and abdominal pain for a couple of days. Her daughter also states that the patient is “not herself”.

Cognitive Status

MMSE 22/30 (performed in the emergency room)

Diagnosis

Physical exam, laboratory data, and imaging data are concerning for diverticulitis and perforation. Surgery was consulted.

**CASE:**

You determine that the patient has capacity to make medical decisions. After patient is given her hearing aids and informed about the risks and benefits of surgery in simple clear language, she agrees to the operation. A partial colectomy with diverting colostomy is done. She has no complications. She has been started on intraveneous (IV) antibiotics, and IV morphine, as needed (prn), has been ordered for pain control. A nasogastric tube (NGT) and indwelling urinary catheter have been inserted. She was transferred to the general medicine/surgical floor and placed in a room near the nursing station.

It is now post-op day 2. This morning, the night float doctor reports that a nurse called him to get a verbal order for a vest and wrist restraints because the patient had pulled out her NGT sometime around 5 am. The patient also refused blood draws this morning. The night float ordered the restraints. On your morning rounds, the nurse tells you that the patient has repeatedly tried to get out of bed and is now tugging on her IV line. The nurse requests that you reinsert the NGT and renew the restraint order now.

You go to the patient’s room. You notice that she is calling out for her husband and her dead mother. You check the nursing chart notes and find out that the patient was lethargic on post-op day 1. The nursing notes also document that she had moderate pain yesterday for which she received morphine IV × 5, has had no bowel movements (BMs) since the surgery, and that her left heel had nonblanchable erythema. You also check the medication list in the electronic medical record and notice that, compared to her pre-admission list, there are new and substituted medications.

*Her medications at this point include:* - glargine insulin 6 units at 6 pm (*type 2 diabetes*) - lispro insulin sliding scale (*type 2 diabetes*) - lisinopril 10 mg daily (*hypertension*) - hydrochlorothiazide 25 mg daily (*hypertension*) - metoprolol 25 mg three times a day (*hypertension*) - simvastatin 20 mg daily (*hyperlipdemia*) - ranitidine 150 mg daily (*ulcer prevention*) - colace 100 mg twice a day (*stool softner*) - bisacodyl suppository daily per rectum (pr) as needed for constipation - morphine sulfate 1–2 mg IV every 4 h as needed for pain - Benadryl (diphenhydramine) 12.5–25 mg IV every 4 h as needed for sleep, itch - lorazepam 1 mg by mouth every 12 h as needed (*anxiety*) - heparin 5000 units subcutaneous q 8 h (*deep vein thrombosis prophylaxis*)	*Her medications prior to admission:* - glipizide XL 10 mg daily (*type 2 diabetes*) - metformin 500 mg twice a day (*type 2 diabetes*) - lisinopril 10 mg daily (*hypertension*) - hydrochlorothiazide 25 mg daily (*hypertension*) - atorvastatin 20 mg daily (*hypercholesterolemia*) - alendronate 70 mg weekly (*osteoporosis*) - calcium w/vitamin D daily (*osteoporosis*) - multivitamin daily - latanoprost one gtt OU at bedtime (*glaucoma*) - timolol one gtt OU twice a day (*glaucoma*) - acetaminophen as needed for pain

Pertinent PE:

Gen—Grimacing in pain. Calling out, “Ma… Ma… help me!”

VS—T 100.6 F (normal 98.6), P 100 (normal 60–80), BP 170/95 (normal 120–140/80–90),

O2 sat 92% on room air (normal 95–100%)

HEENT—Hearing aids are not in place.

Mouth—Mucous membranes are moist.

Neck—No jugular venous distention (JVD)

Lungs—Crackles in both bases.

Heart—Regular rate and rhythm (RRR)

ABD—Soft. Staples are intact, with scant serosanguinous fluid over the incision site. Tenderness around the incision site without warmth or erythema. Otherwise abdomen is non-tender. No bowel sounds.

GU—Urine collection bag is full of light yellow clear urine

EXT—No edema. Left heel has nonblanching erythema and is tender to palpation. Right heel is OK.

NEURO—Moving all of her extremities spontaneously. No focal deficits noted (though she does not cooperate with your attempts to do a full neurological exam).

Pertinent Data:

Chest X-ray (CXR)—atelectasis

Abdominal film—ileus without obstruction

EKG—sinus tachycardia without changes from previous EKG

CPKs—cycle is negative to determine if patient had a heart attack

WBC—12K (normal 4.5–11K), no leftward shift

Hct—31% (normal 36–48%)—blood count

BMP—within normal limits (BUN 25 mg/dL, Cr 1.2 mg/dL)—kidney function

UA—trace leukocyte esterase, RBC 0-2/HPF, WBC 5/HPF, negative for nitrites

**Module III:**

Summary of Hospital Course

Physical exam, laboratory data, and imaging data are concerning for diverticulitis and perforation. Surgery was consulted, and they performed a partial colectomy with diverting colostomy. The patient developed acute delirium post-operatively. On post-op day #2, you went to see the patient after the nurse reported that the patient had removed her NGT, was now in a vest restraint (as ordered by the night float), tried to get out of bed, refused morning blood draws, and tugged on her IV line.

**CASE PART 1:**

Because the patient is acutely agitated and at risk for harming herself, you decide to order haloperidol (Haldol) 0.5 mg IV x one dose. In half an hour, she calms down enough that you are able to reinsert the NGT without difficulty and order diagnostic tests to determine the etiologies of her agitated delirium.

On post-op day 3, she is less confused. PT and OT were unable to begin therapy because she was still delirious.

On post-op day 5, daughter tells you that she is almost back to herself but “not quite”.

During your morning rounds, she appears alert, oriented to person and place but not exact date. On physical exam, incision site is healing well.

PT/OT evaluate her ability to use the bedside commode today. She is able to transfer with the assistance of one person.

By post-op day 6, she is able to ambulate 50 feet in the hallway using a walker. She continues to engage in appropriate conversation with the staff and asks you how her husband is doing. She tolerated a regular diet.

**Outpatient Medications:** glipizide XL 10 mg daily metformin 500 mg twice a day lisinopril 10 mg daily hydrochlorothiazide 25 mg daily atorvastatin 20 mg daily alendronate 70 mg weekly calcium w/vitamin D daily multivitamin daily latanoprost one gtt OU qhs timolol one gtt OU bid acetaminophen prn	**Inpatient Medications:** glargine insulin 6 units at 6 pm lispro insulin sliding scalel isinopril 10 mg daily hydrochlorothiazide 25 mg daily metoprolol 25 mg tid simvastatin 20 mg daily pantoprazole 40 mg daily (after ranitidine was stopped) colace 100 mg bid bisacodyl suppositories prn oxycodone 5 mg qid prn acetaminophen prn Heparin 5000U subcutaneous q 8 h (lorazepam and diphenhydramine were stopped)	**Discharge Medications:** glipizide XL 10 mg daily metformin 500 mg twice a day lisinopril 10 mg dailyhydrochlorothiazide 25 mg daily metoprolol XL 75 mg daily simvastatin 20 mg daily pantoprazole 40 mg daily alendronate 70 mg weekly calcium w/vitamin D daily multivitamin daily latanoprost one gtt OU qhs timolol one gtt OU bid acetaminophen 500 mg QID prn senokot 187 mg twice a day (*constipation*) docusate sodium 100 mg BID (*constipation*)

Physical Exam:

Gen—Alert, attentive.

VS—Afebrile, BP 103/60 (*normal 120–150/80–90*), HR 55 (*normal 60–80*), R 16, O2 sat 98% on room air, pain is now 1/10.

LUNGS—Crackles in both bases.

Heart—RRR.

ABD—Incision site is clean, no erythema or warmth. Colostomy bag contains brown liquid stool. Good bowel sounds. Non-tender, non-distended.

EXT—Left heel stage I pressure sore is non-tender. No eschar or skin breakdown over the left heel.

NEURO—Knows what year it is but states the date and day incorrectly. She tells you she is at University of Louisville Hospital on the sixth floor.

LABS: BMP normal, TSH normal, B12 normal, Hct 32% and stable.

Based on the hospital team’s recommendation and her preference, Mrs. HH is discharged home with skilled services from nursing and physical therapy and surgery clinic follow up. While writing the discharge summary you review the medication list in the electronic medical record and notice that, compared to her pre-admission list, there are new and substituted medications.

The nurse reviews the discharge summary and discharge medications with the patient and her daughter. You send her new prescription medications to the pharmacy electronically. Mrs. HH arrives home on a Saturday.

**CASE PART 2:**

A visiting nurse goes to see the patient at home on Sunday, the day following her hospital discharge. She finds Mrs. HH crying in pain. Her daughter reports that the acetaminophen is not helping. The visiting nurse calls the covering physician and asks for something stronger for pain. The covering physician (who does not know the patient) tells the nurse to send the patient back to the emergency room for evaluation and pain control.
